# Functional Reconstruction of a Hemiglossectomy Defect With an Innervated Musculocutaneous Submental Artery Island Flap

**DOI:** 10.7759/cureus.56835

**Published:** 2024-03-24

**Authors:** Ourania Schoinohoriti, Georgios Mitsopoulos, Evangelos Kalfarentzos, Christos Perisanidis

**Affiliations:** 1 Department of Oral and Maxillofacial Surgery, Dental School, National and Kapodistrian University of Athens, Athens, GRC; 2 Department of Oral and Maxillofacial Surgery, 401 General Military Hospital of Athens, Athens, GRC

**Keywords:** functional tongue reconstruction, mylohyoid nerve, mylohyoid muscle, anterior belly of digastric muscle, innervated submental artery island flap

## Abstract

This report presents the use of an innervated musculocutaneous submental artery island flap (MSAIF) for the functional reconstruction of a hemiglossectomy defect, with the aim of preserving the volume and mobility of the reconstructed tongue to facilitate swallowing and intelligible speech. A 30-year-old male patient diagnosed with T3N0 stage squamous-cell carcinoma of the tongue underwent hemiglossectomy and ipsilateral I-IV selective neck dissection. For reconstruction, an innervated MSAIF with a 9x4 cm skin paddle, including the left submental vessels, ipsilateral anterior belly of the digastric muscle, mylohyoid muscle, and mylohyoid nerve, was harvested and inserted into the tongue defect. Postoperative healing at both donor and recipient sites proceeded without complications.

At a three-year follow-up, the MSAIF has maintained its volume, mobility, and contractility. The patient remains disease-free and reports satisfaction with his swallowing and speech capabilities. The innervated MSAIF represents a reliable and cost-effective reconstruction approach for hemiglossectomy defects, showing favorable results in both swallowing and speech.

## Introduction

Reconstruction of the tongue following tumor resection poses significant challenges, given the intricate muscular anatomy of the tongue and its central function in swallowing, speaking, taste perception, and prevention of aspiration. Various approaches, including direct closure, locoregional flaps, and microvascular free flaps, have been implemented for tongue reconstruction. To functionally reconstruct tongue defects, it is essential to use flaps that restore both the volume and mobility of the neo-tongue [1].

Even in the era of free tissue transfer for head and neck reconstruction, locoregional flaps continue to be popular due to the growing focus on cost-effective treatment, particularly for patients with severe comorbidity, compromised general health, or contraindications to free flap reconstruction. The submental artery island flap (SAIF), which is perfused by the submental vascular pedicle, is an axial pattern flap that has been widely implemented for the reconstruction of facial and intraoral defects. Refinements in the harvesting technique have facilitated pedicle elongation and broadening of the rotation arc, thus extending its indications even for defects of the contralateral upper facial third [2]. In particular, innervated variants of the SAIF have been described for facial reanimation [3].

This article aims to present an alternative technique for the functional reconstruction of a post-resection hemiglossectomy defect, consisting of an innervated musculocutaneous submental artery island flap (MSAIF). The herewith presented technique preserves the volume and mobility of the tongue, facilitating swallowing and intelligible speech and thus improving the patient's quality of life.

This article was previously presented as a meeting abstract at the ‘ICOMS 2023 Vancouver’ on June 11, 2023.

## Case presentation

A 30-year-old male patient with a six-month history of a tongue ulcer on the left side was referred to our department. Histopathological analysis of the incisional biopsy revealed a squamous-cell carcinoma. Magnetic resonance imaging (MRI) showed an irregular soft tissue mass on the left side of the tongue. The patient was staged as T3N0 since no nodal disease was detected through clinical examination or MRI. Oncological resection comprised hemiglossectomy with adequate margins and ipsilateral selective neck dissection at levels I-IV.

**Figure 1:**
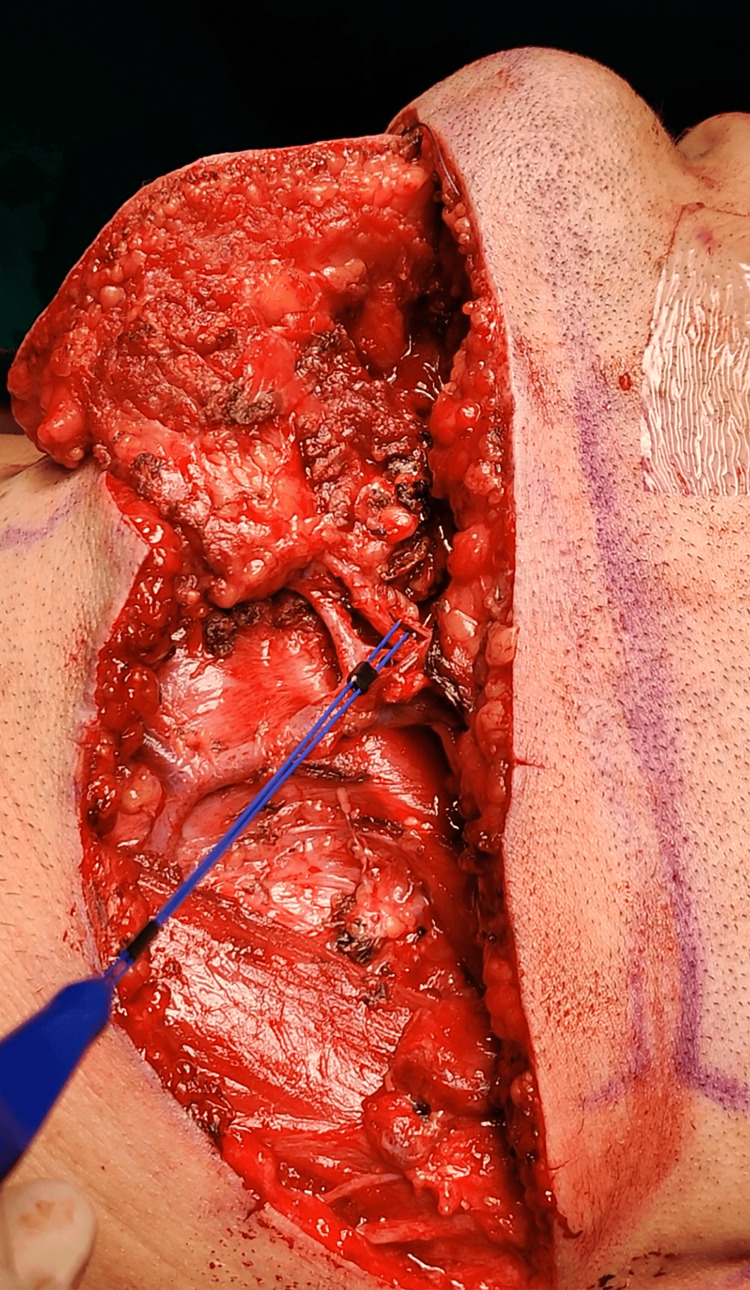
Intraoperative photograph of the patient: the nerve stimulator points to the mylohyoid nerve

An innervated MSAIF was raised in conjunction with concurrently performed neck dissection and comprised a submental skin paddle, the left submental vessels, ipsilateral anterior belly of the digastric muscle (ABDM), mylohyoid muscle, and mylohyoid nerve (Figure 1).

**Figure 2:**
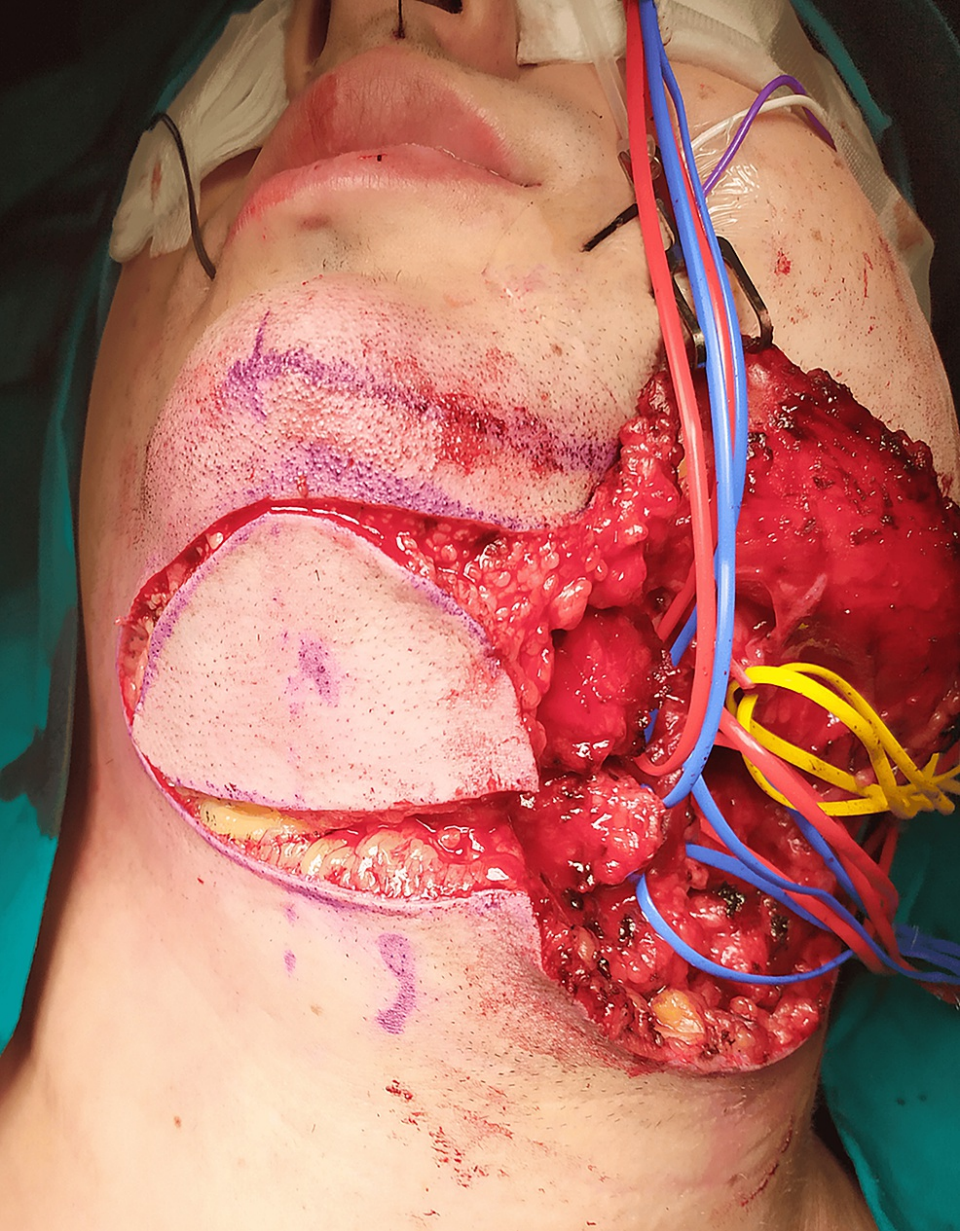
An elliptically shaped 9 x 4 cm submental skin paddle was harvested: the incision was placed superiorly approximately 2 cm below the lower mandibular border and inferiorly following a pinch test, to ensure primary closure of the donor site.

A 9 x 4 cm elliptically-shaped skin paddle was harvested from the submental area. The incision was placed superiorly approximately 2 cm below the lower mandibular border and inferiorly following a pinch test, to ensure primary closure of the donor site (Figure 2).

The flap was raised from the contralateral side in a plane below the mylohyoid muscle while the lymph nodes in the submental triangle were dissected meticulously from the flap. The ipsilateral ABDM was identified and divided at the lower border of the mandible and the intermediate tendon of the digastric muscle. The mylohyoid nerve was identified along the left submental and facial vessels, which were dissected off the submandibular gland. During flap harvesting the innervation of the ABDM and mylohyoid muscle by the mylohyoid nerve was maintained; this was confirmed through contraction of the flap muscles as a response to stimulation of the mylohyoid nerve. The flap was then medially tunneled and inserted into the tongue defect. The donor site was primarily closed in layers. Both the donor and recipient sites healed uneventfully.

**Figure 3:**
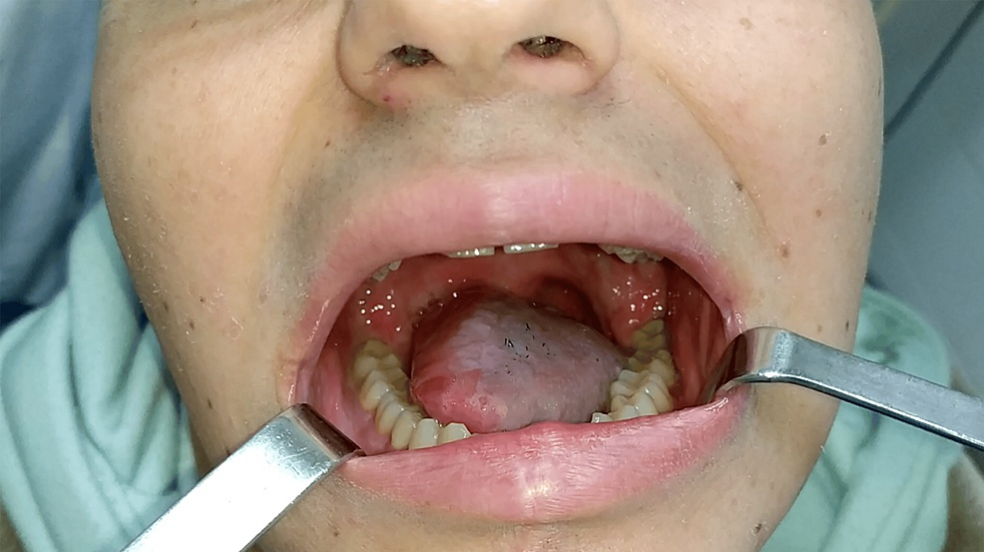
Photograph of the patient at the three-year follow-up, showing that the tongue volume was preserved

The patient received adjuvant radiotherapy up to a total dose of 66 Gy. No volume loss or reduced tongue mobility was observed. At the three-year follow-up examination, the patient shows no evidence of disease, preserves the volume and mobility of the neo-tongue, and remains satisfied with swallowing and speech (Figure 3). His speech was evaluated as clear and intelligible by a speech therapist. The swallow function was evaluated through the EORTC (European Organisation for Research and Treatment of Cancer) QLQ-H&N35 questionnaire: the patient reported no problems when swallowing liquids, pureed or solid food, no choking when swallowing, and no trouble eating; he has also been gaining weight since the completion of radiotherapy, almost retrieving his original BMI. 

## Discussion

The main goals of functional reconstruction of the tongue are to restore and maintain volume, prevent tongue mobility restriction due to scar contraction, and enable tongue movement through dynamically restoring muscular anatomy. Volume restoration facilitates swallowing by establishing the contact of the neo-tongue with the palate and obliterating the oral cavity [1]. Preserving mobility of the reconstructed tongue is closely related to better speech and swallowing outcomes and a higher quality of life [4].

The radial forearm and anterolateral thigh free flaps have been the mainstay for the reconstruction of moderate to large glossectomy defects. However, reconstruction with these non-innervated flaps makes the neo-tongue a rather static structure, resulting in volume loss and mobility limitations. To overcome this concern, dynamic reconstruction with motor-reinervated free flaps was introduced, maintaining muscle tone, volume, and mobility of the neo-tongue [5]. Thus, the innervated musculocutaneous submental artery island flap is a suitable alternative to microsurgical free tissue transfer for the functional reconstruction of tongue defects following cancer resection.

Since its original description by Martin et al. in 1990, several variants of the submental flap have been described, including skin, muscle, submandibular gland, and/or mandibular bone harvesting within the flap, making it adaptable to complex facial and intraoral defects [6]. Faltaous et al. suggested the inclusion of the anterior belly of the digastric muscle in the submental artery island flap to avoid injury of the submental pedicle that passes either deep or superficial to the anterior belly of the digastric muscle [7]. Patel et al. described the inclusion of the mylohyoid muscle in the submental artery island flap to limit the dissection of the submental vascular pedicle to the lateral border of the mylohyoid muscle and protect the perforating branches to the submental skin [8]. In addition, innervated variants of the submental artery island flap have been proposed for facial reanimation procedures. Sakuma et al. reported a case of upper lip reconstruction after tumor resection using a reverse submental flap with a reinnervated anterior belly of the digastric muscle by suturing the mylohyoid nerve to the stump of the buccal branch of the ipsilateral facial nerve [3].

This report presents an innervated musculocutaneous submental artery island flap, used for the functional reconstruction of a hemiglossectomy defect following tumor resection. Voluntary innervation of the transferred flap muscles by the mylohyoid nerve resulted in the contraction of the muscle fibers and maintenance of the muscle tone. Furthermore, innervation of the flap muscles prevented progressive fibrosis and atrophy, thus preserving the volume of the neo-tongue. The skin paddle of the submental flap prevented adhesion to the surrounding tissues during the healing process, thus preserving tongue mobility. In our patient, the preserved contractility, volume, and mobility of the flap proved beneficial in restoring swallowing and intelligible speech.

Compared to microvascular free flaps, the submental artery island flap has the advantage of being harvested from the same operative site without the need for a second surgical team, shortening operative time and hospital stay and reducing overall donor site morbidity. Relative contraindications to this flap include the need for large tissue volume, distant defects, and hair growth at the recipient site. Considerable controversy remains in the literature regarding the oncologic safety of the submental artery island flap for the reconstruction of oral defects following cancer resection, particularly in the setting of metastasis to level I cervical lymph nodes [9]. Proponents of the submental artery island flap have argued that meticulous dissection of the flap allows comprehensive level I dissection and removal of all pathological lymph nodes. Notably, a recent meta-analysis found no significant difference between the submental artery island flap and free tissue transfer in terms of local, regional, and distant recurrence rates [10].

## Conclusions

In conclusion, the innervated musculocutaneous submental artery island flap is herewith described as a suitable alternative to microvascular free tissue transfer for the reconstruction of hemiglossectomy defects following tumor resection. It is reliable and versatile, combining good thickness, contour, and texture match between donor and recipient sites with minimal morbidity and good aesthetic results at the donor site. Voluntary innervation of the flap muscles by the mylohyoid nerve prevents their atrophy and progressive fibrosis, thus preserving the volume and mobility of the reconstructed tongue and maintaining swallowing and intelligible speech.
